# Accessibility of methadone treatment via public transit for syringe services program participants in Miami-Dade County, Florida

**DOI:** 10.21203/rs.3.rs-4791074/v1

**Published:** 2024-08-27

**Authors:** Marina Plesons, Eileen Malecki, Katrina Ciraldo, Emilie Ashbes, Edward Suarez, Hansel E. Tookes, Tyler S. Bartholomew

**Affiliations:** University of Miami Miller School of Medicine: University of Miami School of Medicine; University of Miami Miller School of Medicine: University of Miami School of Medicine; University of Miami Miller School of Medicine: University of Miami School of Medicine; University of Miami Miller School of Medicine: University of Miami School of Medicine; University of Miami Miller School of Medicine: University of Miami School of Medicine; University of Miami Miller School of Medicine: University of Miami School of Medicine; University of Miami Miller School of Medicine: University of Miami School of Medicine

**Keywords:** Methadone, public transit, access, people who inject drugs

## Abstract

Methadone is an opioid receptor agonist medication used in the treatment of opioid use disorder (OUD). Geographic distance to opioid treatment programs (OTPs) is a major barrier to treatment, given requirements for direct observation of dosing and periodic drug screens, and ‘methadone treatment deserts’ are defined as a public transit threshold of 30 minutes. The purpose of this study was to examine public transit access to methadone treatment for participants of a syringe services program (SSP) in Miami-Dade County, Florida. Public transit times were calculated using the R library r5r, which facilitates multi-modal transportation network routing. General Transit Feed Specification data was combined with street network data from OpenStreetMap for Miami-Dade County. Transit times were estimated from the population-weighted centroid of each zip code (n=79) with participants of Miami’s only SSP (n=1597) to the nearest OTP (n=4) using 10 departure windows aligned with OTP service hours. The mean one-way transit time from zip codes with SSP participants in Miami-Dade County to the nearest OTP was 80 minutes. 75 of the 79 (95%) zip codes with SSP participants in Miami-Dade County have a mean transit time to the closest OTP greater than 30 minutes. Transit times differ substantially between zip codes with different numbers of SSP participants, but not between departure windows. Nearly all zip codes with SSP participants in Miami-Dade County can be classified as ‘methadone treatment deserts’. Geographic isolation of methadone treatment from public transit routes represents a significant barrier to equitable OUD treatment.

## Introduction

In 2021, more than 106,000 people in the United States died due to drug overdose, representing a 14% rise in overdose deaths compared to 2020;^1^ of these deaths, approximately 75% involved opioids.^1^ Treatment with a medication for opioid use disorder (MOUD) has been shown to reduce rates of opioid overdose, all-cause mortality, and suicide by more than 50%.^2,3^ Discontinuation of a MOUD, meanwhile, is associated with relapse and overdose; thus the recommended duration of treatment is indefinite and individualized.^4^ However, while the number of facilities providing MOUD and patients receiving MOUD have increased over time,^5^ major gaps remain in the opioid use disorder (OUD) cascade of care.^6^ It is estimated that only 20% of people with OUD in the United States receive specialty care in a given year, less than 35% of those individuals receive evidence-based treatment with a MOUD, and most of those individuals discontinue treatment within months.^6^ Furthermore, significant disparities exist in access to and use of MOUD across the rural-urban continua and by race and ethnicity.^7,8^

Methadone, a full μ-opioid receptor agonist, is one of two opioid receptor agonist medications used in the treatment of OUD. Despite being a life-saving medication, especially for the increasing number of patients who do not tolerate buprenorphine in the era of fentanyl and other high-potency synthetic opioids,^9^ it is tightly regulated by SAMHSA, the DEA, and the FDA as a Schedule II controlled medication. Historically, these regulations required that it be dispensed daily under direct observation at federally and state-designated OTPs until patients met certain criteria, including accrual of time in treatment and demonstrated abstinence from all illicit substances by urine drug testing. After three months of daily observed treatment, OTPs allowed patients with a daily dose ≤ 100 mg two take-home doses per week for the first two treatment years, three take-home doses per week for the third treatment year, and six take-home doses per week for subsequent years, conditioned on appointment attendance and negative urine drug screens.^10^ In addition, patients had to comply with periodic random drug tests and be seen yearly by a physician in the clinic.

In March 2020, to reduce risk of COVID infection for patients and staff at OTPs, SAMHSA made temporary allowances for relaxed take-home protocols, permitting states to allow OTPs to provide 27 take-homes to patients who were stable and 13 take-homes to patients who were less stable. Based on evidence that these relaxed take-home protocols did not result in increased negative outcomes,^11,12^ SAMHSA made these flexibilities permanent in February 2024, marking the first substantial update to the OTP treatment standards in over 20 years. Patients may now be eligible for unsupervised, take-home doses of methadone upon entry into treatment. While this federal change increases access to methadone, states still have the authority to determine their own OTP regulations (see Table 1 for a comparison of previous and current regulations in Florida), and decisions regarding take-home protocols, where permitted, are based on the clinical judgment of healthcare providers.

This regulatory environment stands in stark contrast to that of buprenorphine, a partial μ-opioid receptor agonist also used in the treatment of OUD. In April 2020, SAMHSA removed the requirement for an in-person assessment for the prescription of buprenorphine, thereby enabling new telehealth-based models of care for OUD. Based on evidence that this policy change increased patient engagement and satisfaction with care, it extended this flexibility in the post-pandemic era.^14^ Likewise, in December 2022, US Congress removed the requirement for practitioners to apply for a special waiver prior to prescribing buprenorphine for OUD. Since then, there has been progress in expanding low-threshold buprenorphine treatment in critical settings like SSPs.^15^ However, evidence demonstrates that White patients are four times more likely to be prescribed buprenorphine as Black patients,^16^ and that buprenorphine patients are more likely than methadone patients to be employed, have higher levels of education, and use prescription opioids instead of heroin.^17^ Thus, this progress risks exacerbating existing racial and socioeconomic inequities in treatment for OUD unless paired with similar efforts to create low-threshold methadone treatment.^18^

Availability and accessibility, two of the five dimensions of access to healthcare, of OTPs are critical considerations in increasing initiation and retention in methadone treatment.^19^ Research has shown that a significant number of patients travel considerable distances to access methadone treatment at OTPs.^20^ Geographic distance to OTPs, and the associated time and financial costs of attending treatment, are thus major barriers that people who inject drugs (PWID), including those enrolled in syringe services programs (SSPs), face in accessing methadone.^21,22^ As a result, studies have also shown that geographic distance is negatively associated with methadone treatment retention.^23–25^ However, nearly all studies examining the accessibility of OTPs have utilized geographic distance or driving time, rather than public transportation transit time.^20,22,23,26–30^ This is an important distinction since lower-income individuals, especially those in urban areas, are more likely to rely on public transportation,^31^ and access to efficient, well-functioning transportation systems is a notable social determinant of health equity.^32^

In 2021, approximately 7,800 deaths due to drug overdose occurred in Florida^33^. However, as of October 2023, there were just 78 clinics licensed to dispense methadone for the treatment of OUD in Florida and 4 in Miami-Dade County. To date, there are no studies investigating the geographic accessibility of methadone treatment in either the state or the county. To begin to fill this gap, this study sought to examine public transit access to methadone treatment for participants of an SSP in Miami-Dade County, Florida.

## Methods

Based on the methods utilized by Yücel et al,^26^ this study leveraged open-source geographic databases and local public transit scheduling data to estimate multi-modal public transit access to methadone treatment for SSP participants in Miami-Dade County. We then compared estimated transit times to the 30-minute transit threshold established in the literature as an indication of a ‘methadone treatment desert’^34^ to describe methadone treatment accessibility.

### Data Sources

Four data sources were required for this analysis. First, OTPs operating in Miami-Dade County as of October 2023 were identified from SAMHSA’s Opioid Treatment Program Directory and contacted to confirm provision of outpatient methadone treatment. The addresses of these OTPs were obtained from the Florida Department of Children and Families and converted into latitude and longitude coordinates, as were the addresses of the SSP’s fixed site and mobile locations. Second, the zip codes of PWID enrolled as participants of the SSP (n = 2267 as of October 30, 2023, n = 1597 of whom reported a zip code in Miami-Dade County on enrollment) were extracted from the SSP’s administrative data. Of the 89 zip codes in Miami-Dade County, 79 had at least one SSP participant; zip codes with ≤ 5 individuals were suppressed to maintain participant anonymity. The population-weighted centroids of these zip codes were identified from the Department of Housing and Urban Development. Third, street network data for Miami-Dade County was obtained from OpenStreetMap, a spatial data source that provides a free, maintainable, and editable map of the world. Finally, General Transit Feed Specification data was obtained from the Miami-Dade Department of Transportation and Public Works. General Transit Feed Specification data is published by transit agencies and contains the locations of transit stops and the schedules and routes of the various transit modes in a transit network for use by a wide variety of software applications.

### Routing Analysis

Following the methods proposed by Yücel et al,^26^ this routing analysis utilized the R library r5r, which facilitates multi-modal transportation network routing.^35^ The General Transit Feed Specification and OpenStreetMap data were combined to create the transit network. The population-weighted centroids of each zip code with PWID enrolled in the SSP were designated as points of departure, and the locations of the four OTPs in Miami-Dade County were designated as points of interest. Finally, ten departure windows were specified in line with the OTPs’ service hours (8am and 10am Monday-Friday). The r5r library then identified the nearest point of interest for each point of departure and calculated the average one-way transit time for each departure window.

### Threshold Analysis and Visualization

Based on the definition established by Hyder et al,^34^ we utilized 30 minutes as the threshold above which a zip code was classified as being a ‘methadone treatment desert.’ Geographic Information System (GIS) maps were then constructed to support visualization of the distribution of SSP participants in Miami-Dade County and average transit times by zip code.

## Results

An SSP participant in Miami-Dade County had a mean 80-minute one-way transit time to their nearest OTP. Assuming daily round trips 6 days per week (which excludes Sundays when the four OTPs are closed and patients receive a take-home dose), individuals would have to travel approximately 16 hours per week to remain engaged in methadone treatment. Transit times did not differ substantially between departure windows, ranging from 79 to 81 minutes (Table 2). However, they differed substantially between zip codes with various numbers of SSP participants, ranging from a minimum of 66 minutes from zip codes with 15–31 SSP participants to a maximum of 98 minutes from zip codes with ≤ 5 SSP participants (Table 3). For the zip code with the highest number of SSP participants (≥ 75), the mean transit time was 70 minutes.

**Figure 1:**
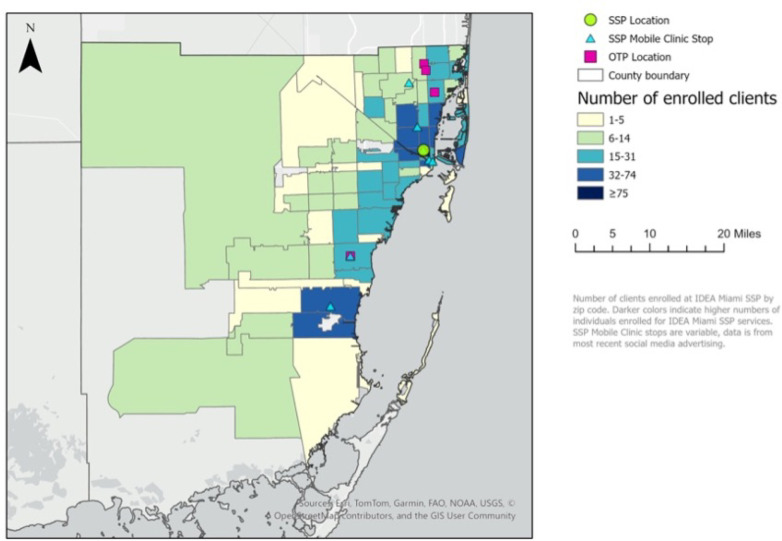
Distribution of SSP participants in Miami-Dade County

**Figure 2:**
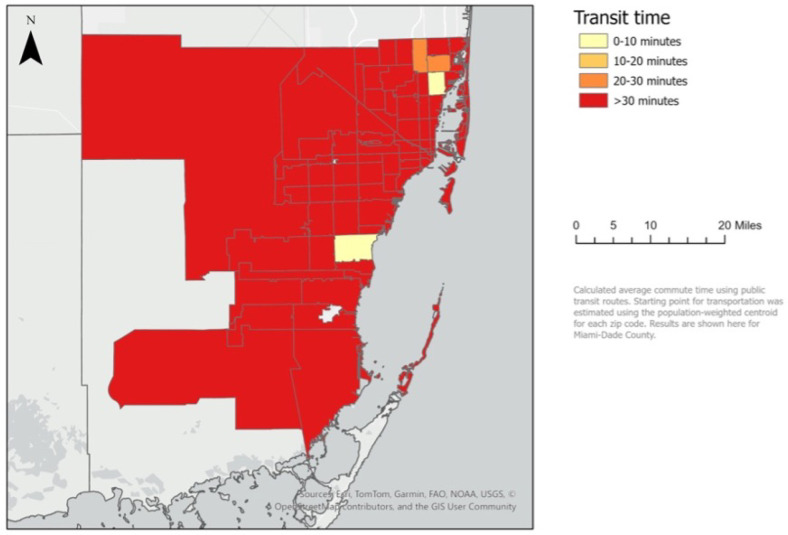
Average transit times to the closest OTP by zip code in Miami-Dade County

The distribution of SSP participants in Miami-Dade County and mean transit times by zip code are mapped in Figs. 1 and 2, respectively. Of the 79 zip codes with SSP participants in Miami-Dade County, 75 of them (95%) had a mean transit time to the closest OTP greater than 30 minutes, thus categorizing them as ‘methadone treatment deserts’. Of the 4 zip codes with mean transit times less than or equal to 30 minutes, one had 6–14 participants and three had 15–31 SSP participants.

## Discussion

We identified extremely long one-way public transit times during OTP service hours for PWID enrolled in the county’s only SSP, with an average transit time of 80 minutes. Using the established definition of a ‘methadone treatment desert’ as a public transit time of more than 30 minutes,^34^ nearly all (95%) of the zip codes with SSP participants were classified as living in a ‘methadone treatment desert’. Unlike Yücel et al’s findings, and those of analyses of public transit access to other health services in a handful of cities around the world,^26^ transit times did not differ substantially between departure windows. However, they did differ between zip codes with various numbers of SSP participants. While transit times were greatest for zip codes with ≤ 5 SSP participants, they were all still more than double the 30-minute threshold for all categories.

To our knowledge, only one other study has examined public transit times to methadone treatment in the United States; this study was conducted in Franklin County, Ohio and identified a substantially shorter median transit time of 42 minutes.^34^ Internationally, one other study, on which the methods of this analysis is based, quantified public transit times to methadone treatment in Toronto, Canada; this study, likewise, found significantly greater access to methadone treatment with an average transit time of only 24 minutes.^26^ However, the findings of this study align with more general findings regarding driving times to methadone treatment, which have demonstrated that many patients travel considerable distances to access methadone treatment at OTPs.^8,20,22,23,27^

While regulatory changes in recent years have enabled important progress in expanding low-threshold buprenorphine treatment through novel models of care (e.g., telehealth) in new settings (e.g., SSPs),^15,21^ we risk exacerbating existing racial and socioeconomic inequities in treatment for OUD unless similar progress is made for methadone treatment.^18^ In the Miami-Dade County context, this analysis points to a need for improvements in public transit generally, establishment of additional OTPs, creation of mobile medication units, and use of exception requests that can be submitted to SAMHSA on an individual basis to permit additional take-home doses. Notably, the zip code with the highest number of SSP participants contains the county’s public safety net hospital and is adjacent to the zip code with the county’s Veterans Affairs Medical Center, both of which would be logical candidates for additional OTPs. At the state and federal level, there is a need for additional policy revisions to reduce broader barriers to initiation of methadone treatment and improve retention – geographic and otherwise.

This study has a few notable limitations. First, because of our focus on the accessibility of methadone treatment for SSP participants by public transit in Miami-Dade County, Florida, we did not consider accessibility of methadone treatment for people with OUD, more generally. Second, the zip code locations in the SSP’s database are those reported by SSP participants at the time of their enrollment in the program. It is possible that their locations may have changed in the time since then; this is especially true for people experiencing homelessness given the frequency with which they change locations, both by choice and due to police intervention. Finally, we estimated transit times from the population-weighted centroids of zip codes with SSP participants. As noted by Yücel et al,^26^ individuals who do not live at this centroid likely experience slightly different transit times than those calculated in the analysis. Despite these limitations, our analysis suggests the need for urgent action to 1) advance federal and state regulations for low-threshold methadone treatment, 2) capitalize fully on present opportunities, e.g., by establishing additional OTPs and mobile medication units and by increasing utilization of exceptions for take-home doses, and 3) improve public transit in Miami-Dade County.

## Conclusion

The public transit times between the locations of SSP participants in Miami-Dade County and the county’s four OTPs represent a significant barrier to methadone treatment initiation and retention. While the recent relaxation of take-home protocol regulations represents a significant positive development, access to methadone remains inadequate to meet the demand of people with OUD. Geographic isolation of methadone treatment from public transit routes represents a significant and unmitigated barrier to equitable OUD treatment.

## Figures and Tables

**Table 1:** Current and previous take-home allowances for methadone treatment in Florida^13^

**Pre COVID-19 pandemic**	**Take-home privileges shall be limited to the following:** **i. No take-homes shall be permitted during the first 30 days following placement unless approved by the state authority.** **ii. Following 30 consecutive days in treatment, the client may be eligible for 1 take-home per week from day 31 through day 90, provided that the client has had negative drug screens for the preceding 30 days.** **iii. Following 90 consecutive days in treatment, the client may be eligible for 2 take-homes per week from day 91 through day 180, provided that the client has had negative drug screens for the preceding 60 days.** **iv. Following 180 consecutive days in treatment, the client may be eligible for 3 take-homes per week with no more than a 2-day supply at any one time from day 181 through 1 year, provided that the client has had negative drug screens for the preceding 90 days.** **v. Following 1 year in treatment, the client may be eligible for 4 take-homes per week with no more than a 2-day supply at any one time through the second year of treatment, provided that the client has had negative drug screens for the preceding 90 days.** **vi. Following 2 years in treatment, the client may be eligible for 5 take-homes per week with no more than a 3-day supply at any one time, provided that the client has had negative drug screens for the preceding 90 days.**

**Current (as of May 19, 2022)**	Take-home privileges shall be limited to the following: i. During the first 90 days of treatment, the take-home supply is limited to a single dose each week. The individual shall ingest all other doses under appropriate medical supervision. ii. In the second 90 days of treatment, the take-home supply is limited to two doses per week. iii. In the third 90 days of treatment, the take-home supply is limited to three doses per week. iv. In the remaining months of the first year, an individual may be given a maximum of six-day supply of take-home medication. v. After one year of continuous treatment, an individual may be given a maximum two-week supply of take-home medication. vi. After two years of continuous treatment, an individual may be given a maximum of one-month supply of take-home medication but must make monthly visits.

**Table 2:** Mean transit times by departure window

Day	Time	Transit time (minutes)			
		Mean	Std Dev	Min	Max
Monday	8am	79.14	30.77	7	153
	10am	80.62	31.72	7	153
Tuesday	8am	79.14	30.77	7	153
	10am	80.62	31.72	7	153
Wednesday	8am	79.14	30.77	7	153
	10am	80.62	31.72	7	153
Thursday	8am	79.14	30.77	7	153
	10am	79.17	30.89	7	153
Friday	8am	80.60	31.60	7	153
	10am	79.17	30.89	7	153

**Table 3:** Mean transit times by number of SSP participants (n = 1597) by zip code (n = 77)

Number of SSP participants per zip code	N	Transit time (minutes)			
		Mean	Std Dev	Min	Max
≤ 5	20	98.07	32.25	48	153
6–14	25	79.52	31.20	22	127
15–31	21	65.94	27.24	7	112
32–74	10	73.60	18.29	48	98
≥ 75	1	69.80	-	70	70

## Data Availability

Data and materials are available upon request to the corresponding author.

## Abbreviations

COVID-19: Coronavirus disease 2019
DEA: Drug Enforcement Administration
FDA: Food and Drug Administration
GIS: Geographic Information System
MOUD: Medication for opioid use disorder
PWID: People who inject drugs
OTP: Opioid treatment program
OUD: Opioid use disorder
SAMHSA: Substance Abuse and Mental Health Services Administration
SSP: Syringe services program
